# Transactions of the Buffalo Obstetrical Society

**Published:** 1890-03

**Authors:** William C. Krauss

**Affiliations:** Secretary, Editor of the Transactions


### TRANSACTIONS OF THE BUFFALO OBSTETRICAL SOCIETY.

Reported by WILLIAM C. KRAUSS, M. D., Secretary, Editor of the Transactions.

### STATED MEETING, JANUARY 28, 1890.

THE Vice-President, Dr. C. C. FREDERICK, in the chair.

The following-named were elected officers for the ensuing year:

*President*, Dr. C. C. FREDERICK.

*Vice-President*, Dr. A. H. BRIGGS.

*Secretary and Treasurer*, Dr. WM. C. KRAUSS.

Dr. KRAUSS was also elected Editor of the Transactions.

Dr. W. W. POTTER read a paper, entitled,

### A LATER STUDY OF PELVIC INFLAMMATIONS.

The object of the paper was to bring forward the expressed views of the essayist to meet the modern pathological teachings on the subject, and to discuss it in the rays of its latest light.

Due credit was given to Emmet for the great work he had done, but his doctrine of pelvic cellulitis was not accepted as a satisfactory explanation of the phenomena of pelvic inflammations. Bennet's doctrine of cervicitis was referred to as indicating the extreme views of his period, which had now become obsolete. The trend of the present pathology was to consign the so-called pelvic cellulitis to a similar oblivion.

The terms peri- and parametritis were misleading as to pathology, and hence deceptive as to management. The pathology of Bernutz and Goupil, now nearly thirty years old, had not been much improved upon. They recognized that the peritoneum was generally the seat of a sympathetic inflammation that had its origin in the Fallopian tubes, or ovaries, or both. This is what is being admitted to-day by the most competent observers, and it will simplify the whole subject, make it clearer to the student, and result in greater relief to the patient.

Three principal groups of pelvic inflammation were named, as follows:

1. Those of puerperal origin.
2. Those of gonorrheal origin.
3. Those of traumatic origin.

Under the latter were included all infections carried to the endometrium by unclean instruments, tents, or medicinal agents (McMurtry), and those arising from traumatism in any way.

DISCUSSION.

Dr. M. Hartwig.—The original division of the inflammatory conditions of the pelvic cavity into para- and perimetritis and cellulitis was proposed by Virchow. Speaker does not believe now he could speak of parametritis as occurring so frequently, and yet, at the present time, it seems a rather infrequent affection. Thinks that parametritis occurs often after confinement, and results in an abscess on one side. If parametritis exists as the main affection, the surrounding tissue will soon become inflamed, especially the broad ligament.

Dr. Ingraham.—Does not believe that pelvic peritonitis is more frequent than pelvic cellulitis.

Dr. P. W. Van Peyma.—The question as to whether all formations of pus comes from infection from without, is the most important one in the discussion of this subject. Does not believe the older men were mistaken in regard to the frequency of parametritis.

Dr. W. S. TREMAINE.—Inflammations are the same everywhere, being modified only by the tissues in which they reside, as in mucous, serous, or cellular tissue. It is important to know whether the inflammation is in or beneath the serous membrane. Has seen many cases of parametritis, and thinks it a frequent and most common occurrence. Has opened abscesses which were due to a cellular infiltration and inflammation. Inflammations occurring in the peritoneum are simular to those in the pleural cavity, and does not believe that all inflammations of the peritoneal cavity are due to a gonorrheal or septic cause. Speaker dissents from the so-called accepted views, that most of the pelvic inflammations are due to gonorrheal infection. Has seen pelvic inflammations in girls under sixteen years of age, and knows that they were free from gonorrhea, although they may have had a sero-purulent discharge. The middle course, between Tait and Emmet, is the safer one to pursue.

Dr. W. C. KRAUSS.—Inasmuch as the peritoneal cavity resembles the pleural, both being lined with a serous membrane, the inflammatory processes of both must be more or less similar. The peritoneal cavity, being supplied with large amounts of cellular tissue, and the ready manner with which septic germs enter it, render it susceptible to certain forms of inflammation not found in the thorax. Does not believe that all inflammations of the pelvis are due to a septic germ, but that there may be inflammatory processes, with presence of leucocytes not due to micro-organisms. Believes that the gonococci are productive of the majority of the pelvic inflammatory conditions.

Dr. A. H. BRIGGS.—The general practitioner does not draw lines as closely as the specialist. To the former, the important question lies in the treatment.

Dr. C. C. FREDERICK believes that there are true cases of parametritis following abortion with septic infection, causing inflammation; these have gone on forming abscesses, and yet no peritonitis was present.

Dr. POTTER, closing the discussion, said he had very little to add, inasmuch as there had been no arguments to overthrow the position taken in the paper. Virchow announced his pathology of pelvic cellulitis many years ago, and had not revised it to agree with later investigations. Moreover, Virchow was not a gynecologist, and has not had opportunities to judge of the condition of the organs of the pelvis in the living as revealed by abdominal section. When Dr. Tremaine asserts that he does not believe in the gonorrheal origin of salpingitis, and that it is contrary to his experience, it is presumed that he has failed to find the gonococus. This must be accepted in such cases as have been examined microscopically, but not otherwise.

